# Early identification of high-risk myelosuppression subtypes in DLBCL patients undergoing R-CHOP chemotherapy

**DOI:** 10.3389/fmed.2026.1839446

**Published:** 2026-08-18

**Authors:** Keyi Yang, Tianming Du, Guoxin Liu, Chen Li, Qiang Kang, Xinglong Zhang, Yang Hong, Na Sun, Wanqiang Ma, Fanli Kong, Guangjun Fan

**Affiliations:** 1 Department of Pharmacy, The Second Affiliated Hospital of Dalian Medical University, Dalian, Liaoning, China; 2 Department of Pulmonary and Critical Care Medicine, Shengjing Hospital of China Medical University, Shenyang, China; 3 College of Medicine and Biological Information Engineering, Northeastern University, Shenyang, China; 4 The 967th Hospital of PLA Joint Logistics Support Force, Dalian, China; 5 Department of Radiology, Xing’an League People’s Hospital of Inner Mongolia, Inner Mongolia, China; 6 Department of Hematology, The Fourth Affiliated Hospital of China Medical University, Shenyang, Liaoning, China; 7 Department of Pharmacy, Central Hospital of Dalian University of Technology, Dalian, China

**Keywords:** chemotherapy-induced myelosuppression, DLBCL, longitudinal dynamics, predictive biomarkers, sub-phenotyping

## Abstract

**Background:**

Despite prophylactic interventions, chemotherapy-related myelosuppression remains an important clinical concern in patients with diffuse large B-cell lymphoma (DLBCL) receiving R-CHOP. This study characterized its occurrence patterns, baseline predictors, temporal risk, and dynamic heterogeneity.

**Methods:**

We established an 8-year longitudinal cohort of patients with DLBCL who received standard-dose R-CHOP between 2016 and 2024. Endpoints included WHO-defined grade ≥1 and grade ≥3 myelosuppression and severe lineage-specific complications. Correlation analyses evaluated associations between baseline laboratory parameters and HGB-, NEUT-, PLT-, and WBC-related suppression. Multiple machine-learning models were developed for each endpoint and hematologic dimension. L2-regularized Cox proportional hazards models were used for time-to-event analyses, with supplementary time-dependent Cox analyses evaluating G-CSF exposure for NEUT-related endpoints. K-means clustering of longitudinal hematologic features was performed to identify dynamic subphenotypes.

**Results:**

Among 262 patients, advanced Lugano stage and the high-risk IPI subgroup were associated with several severe myelosuppression outcomes, while sex, infection status, and functional or anthropometric characteristics showed endpoint-specific associations. HCT, HGB, and RBC were negatively associated with HGB- and PLT-related myelosuppression and grade ≥3 NEUT-related myelosuppression, whereas RDW-CV and RDW-SD were positively associated with several severe outcomes. Models for HGB- and PLT-related endpoints achieved AUCs >0.80 for grade ≥1, grade ≥3, and corresponding severe-complication outcomes, whereas NEUT-related models showed lower discrimination. Baseline HCT was independently associated with lower hazards of grade ≥1 HGB-related myelosuppression (HR = 0.918, 95% CI 0.894–0.943), grade ≥3 HGB-related myelosuppression (HR = 0.910, 95% CI 0.882–0.940), and severe anemia (HR = 0.934, 95% CI 0.902–0.966). RDW-CV independently increased the hazards of grade ≥1 and grade ≥3 PLT-related myelosuppression, while baseline NEUT# reduced the hazard of grade ≥1 NEUT-related myelosuppression (HR = 0.835, 95% CI 0.760–0.918; FDR-adjusted *p* = 0.0026). G-CSF exposure was not significantly associated with NEUT-related endpoints after endpoint-level FDR correction. Clustering identified a high-severity, high-variability subtype and a comparatively mild, stable subtype; recovery patterns differed across hematologic dimensions.

**Conclusion:**

R-CHOP-related myelosuppression is heterogeneous in occurrence, temporal evolution, and recovery. Baseline clinical and laboratory features may support early risk stratification, dynamic monitoring, and individualized supportive care.

## 1 Background

Diffuse large B-cell lymphoma (DLBCL) is the most common aggressive subtype of adult non-Hodgkin lymphoma (NHL), accounting for approximately 30–40% of all cases and imposing a substantial global epidemiological burden (1–4). DLBCL originates from mature B cells and typically presents with rapidly progressive lymphadenopathy and multi-organ involvement (5). Currently, the anti-CD20 monoclonal antibody rituximab combined with cyclophosphamide, doxorubicin, vincristine and prednisone (R-CHOP) constitutes the standard first-line immunochemotherapy for DLBCL, significantly improving cure rates and long-term survival (6, 7). However, several agents within this regimen, particularly cyclophosphamide and doxorubicin, cause myelosuppression as an adverse effect while exerting antitumor activity, creating a clinical tension between therapeutic benefit and toxicity risk (8, 9). Cyclophosphamide induces tumor cell apoptosis by DNA cross-linking but also damages rapidly proliferating hematopoietic stem and progenitor cells (10, 11). Doxorubicin exerts marrow toxicity via topoisomerase II inhibition and generation of reactive oxygen species that injure DNA (12, 13). Such direct injury to hematopoietic stem cells can produce severe dose-limiting toxicity, clinically manifesting as leukopenia, neutropenia (incidence 30–40%), anemia and thrombocytopenia (14–16). These cytopenias increase the risk of infection and bleeding and may necessitate treatment delays or dose reductions, thereby adversely affecting therapeutic efficacy and long-term prognosis (17–19).

In clinical practice, granulocyte-colony stimulating factor (G-CSF) is commonly used to prevent neutropenia and maintain chemotherapy dose intensity (20), while growth factors and blood transfusions are employed to manage anemia and thrombocytopenia. However, not all patients respond well to these supportive measures, and even with widespread use, the manifestations of myelosuppression exhibit substantial interindividual variability (21–23). Some patients develop rapidly progressive severe cytopenias early in chemotherapy, rendering them unable to tolerate standard chemotherapy regimens and accompanied by complications such as severe anemia, bleeding, and infections; whereas other patients exhibit a dynamic recovery pattern, in which blood counts nadir and then spontaneously recover (24). Moreover, these medications have additional side effects; for example, while G-CSF shortens the duration of neutropenia, it may cause bone pain, recurrence of febrile neutropenia, and even pose a potential risk of marrow exhaustion (25). Blood product transfusion carries risks of infection, allergic reactions and alloimmunization (26). Therefore, analyzing the influence of baseline characteristics on myelosuppression, exploring the extent to which it is affected by bone marrow reserve and treatment course, and identifying patients requiring closer follow-up monitoring are of particular importance during R-CHOP treatment.

This study aimed to systematically evaluate the occurrence patterns, risk characteristics, and potential heterogeneity of myelosuppression during R-CHOP treatment in patients with DLBCL. First, this study analyzed the correlations between baseline clinical characteristics, laboratory parameters, and myelosuppression to evaluate their potential as biomarkers. On this basis, machine learning models were further constructed to assess predictive performance, while model performance was additionally used to explore whether different types of myelosuppression were more likely to be influenced by patients’ baseline hematopoietic reserve status or by subsequent treatment processes. Subsequently, incorporating the time dimension, Cox regression models were established to systematically evaluate the risk of occurrence of different types and severities of myelosuppression, in order to quantify the risk contributions of baseline clinical and laboratory characteristics. Finally, exploratory analyses were performed using unsupervised clustering and related methods to investigate the existence of distinct myelosuppression subtypes and to further analyze the relationships between these subtypes and the occurrence, severity, and clinical outcomes of myelosuppression.

## 2 Methods

### 2.1 Study population

To characterize the myelosuppression patterns in patients with diffuse large B-cell lymphoma (DLBCL) undergoing long-term R-CHOP therapy, an 8-year longitudinal cohort (2016–2024) was established at Shengjing Hospital of China Medical University. Data were extracted and organized based on the hospital’s electronic medical record system. Regarding subject selection, inclusion criteria were as follows: (1) age ≥ 18 years; (2) pathologically confirmed DLBCL; (3) receipt of standard-dose R-CHOP without treatment modification; (4) availability of complete laboratory monitoring data; and (5) completeness of clinical data and follow-up information. Exclusion criteria included: (1) coexistence of other malignancies; (2) active infection, immunodeficiency, or autoimmune disease; (3) pre-existing myelosuppression or severe bone marrow dysfunction; (4) cases diagnosed as relapsed/refractory at initial presentation; (5) clear evidence from medical record review of prior indolent lymphoma history or clinical/pathological evidence suggesting transformed DLBCL; and (6) missing key data. This study was approved by the Ethics Committee (approval No. 2023PS1107K), and the requirement for informed consent was waived. All data were uniformly de-identified.

### 2.2 Data acquisition and quality control

Clinical data collected from the included patients included sex, age, height, weight, BMI, diabetes mellitus, hypertension, IPI score, Lugano stage, bone marrow involvement, subgroup, germinal center subtype, ECOG score, and infection status. Baseline laboratory parameters included in the analysis were lactate dehydrogenase (LDH), albumin/globulin ratio (A/G), albumin (ALB), alkaline phosphatase (ALP), alanine aminotransferase (ALT), aspartate aminotransferase (AST), basophil percentage (BASO%), calcium ion (Ca), cholinesterase (CHE), creatine kinase (CK), creatine kinase-MB (CK-MB), chloride ion (Cl^−^), cystatin C (Cys-C), direct bilirubin (DBIL), eosinophil absolute count (EO), gamma-glutamyl transferase (GGT), bone marrow cell proportion, absolute bone marrow cell count, hematocrit (HCT), hemoglobin (HGB), indirect bilirubin (IDBIL), potassium ion (K^+^), lymphocyte percentage (LYMPH%), mean corpuscular hemoglobin (MCH), mean corpuscular hemoglobin concentration (MCHC), mean corpuscular volume (MCV), monocyte absolute count (MONO#), mean platelet volume (MPV), neutrophil absolute count (NEUT), nucleated red blood cell absolute count (NRBC), platelet-large cell ratio (P-LCR), plateletcrit (PCT), platelet distribution width (PDW), platelet count (PLT), red blood cell count (RBC), red blood cell distribution width-coefficient of variation (RDW-CV), red blood cell distribution width-standard deviation (RDW-SD), white blood cell count (WBC), and creatinine (CREA). Included patients were required to have a follow-up duration of at least 21 days. The longitudinal sampling time points included in this study comprised blood samples collected during hospitalization as well as blood samples obtained between two chemotherapy cycles. As shown in Table 1, Myelosuppression grading was based on WHO standards, focusing on neutropenia (NEUT), anemia (HGB), thrombocytopenia (PLT), and leukopenia (WBC).

**Table 1:** WHO grading criteria for chemotherapy-induced myelosuppression.

Grade	WBC (×10^9^/L)	NEUT (×10^9^/L)	PLT (×10^9^/L)	HGB (g/L)
0	≥4.0	≥2.0	≥100	≥110
1	3.0 ~ 3.9	1.5 ~ 1.9	75 ~ 99	95 ~ 109
2	2.0 ~ 2.9	1.0 ~ 1.4	50 ~ 74	80 ~ 94
3	1.0 ~ 1.9	0.5 ~ 0.9	25 ~ 49	65 ~ 79
4	<1.0	<0.5	<25	<65

For baseline stratified comparisons, patients were categorized into low- and high-risk subgroups according to the International Prognostic Index (IPI), with IPI scores of 0–2 classified as the low-risk subgroup and scores of 3–5 classified as the high-risk subgroup.

To systematically evaluate the risk of chemotherapy-related myelosuppression, this study established three levels of clinical labels according to different severities and clinical consequences: (1) occurrence of grade ≥1 myelosuppression; (2) occurrence of grade ≥3 myelosuppression; and (3) occurrence of severe myelosuppression-related complications, including HGB < 60 g/L (severe anemia), NEUT < 0.5 × 10^9^/L (severe neutropenia with high infection risk), and PLT < 20 × 10^9^/L (high bleeding risk).

### 2.3 Risk stratification and prediction of myelosuppression

According to the above research objectives and different levels of endpoints, this study first analyzed the associations between baseline clinical characteristics, laboratory parameters, and myelosuppression to evaluate their potential as biomarkers and risk stratification indicators. Depending on whether variables conformed to a normal distribution, Pearson or Spearman correlation analyses were performed to systematically assess the relationships between laboratory parameters and different hematologic suppression dimensions (HGB, NEUT, PLT, and WBC).

On this basis, to further evaluate the predictive value of baseline indicators for myelosuppression occurrence and to explore from a modeling perspective whether different types of myelosuppression were more likely to be influenced by patients’ baseline hematopoietic reserve status or by subsequent treatment processes, this study constructed artificial intelligence predictive models based on myelosuppression occurrence labels. Independent predictive models were established for different levels of endpoints, while separate modeling analyses were also performed for each type of myelosuppression event. During model construction, multiple machine learning and neural network approaches were compared, including linear models, kernel methods, distance-based classifiers, discriminant analysis, decision trees, ensemble learning models, and multilayer perceptrons. All models were developed under a unified data preprocessing framework, with feature standardization performed according to the characteristics of different algorithms. Hyperparameter optimization was conducted using a grid search strategy. To address class imbalance, class-weighting methods were introduced during model training, and the dataset was divided into training and validation sets at a ratio of 8:2. Finally, model performance was comprehensively evaluated using multiple discriminative metrics in the training and validation/test sets to identify predictive models with good stability and discriminative ability.

For each endpoint, this study calculated the total sample size, number of events, number of non-events, and event rate, and applied a single-population proportion sample size estimation method based on the event rate to calculate the theoretically required sample size. This method comprehensively considered the event rate, confidence level, and allowable error to evaluate whether the current sample size could stably reflect the population event distribution. The confidence level was set at 95%, and the allowable error was set at 0.07. Subsequently, the actual included sample size was compared with the theoretically required sample size to evaluate whether the current dataset satisfied the sample size requirements for cross-sectional analysis. When the actual sample size was greater than or equal to the theoretically required sample size, the sample size was considered adequate; otherwise, it was considered insufficient.

### 2.4 Time-to-event risk analysis of myelosuppression

On the basis of cross-sectional risk assessment and predictive model construction for myelosuppression occurrence, this study further introduced time variables for risk evaluation in order to characterize the temporal occurrence patterns and risk evolution features of different types of myelosuppression. Greater emphasis was placed on “when myelosuppression occurs” and on the dynamic effects of different baseline characteristics on event risk. Within a time-to-event framework, the risk contributions of baseline clinical and laboratory indicators to different types and severities of myelosuppression were quantified, thereby providing a foundation for subsequent exploration of the dynamic heterogeneity of myelosuppression.

For different levels of myelosuppression endpoints (grade 1 myelosuppression, grade 3 myelosuppression, and severe clinical complications), separate time-to-event analysis models were established to systematically evaluate the effects of baseline clinical and laboratory characteristics on the occurrence risk of different myelosuppression outcomes. During model construction, the time to first occurrence of each type of myelosuppression event was defined as the survival time, and the occurrence status of the corresponding event was used as the outcome variable to construct univariable and multivariable Cox proportional hazards regression models. Included variables comprised baseline clinical characteristics and baseline laboratory parameters. To reduce the impact of multicollinearity on model stability, highly correlated variables were first filtered based on the correlation matrix. When the absolute value of the correlation coefficient between variables exceeded 0.85, only one variable was retained for subsequent analysis. Subsequently, univariable Cox analyses were performed for all variables, and variables with *p* < 0.1 were selected as candidate variables for inclusion in the multivariable model. During the multivariable analysis stage, a Cox proportional hazards model with an L2 penalty term (penalizer = 0.1) was applied to improve model robustness and reduce the risk of overfitting. Model results were presented as hazard ratios (HRs) and 95% confidence intervals (95% CIs), and the Benjamini–Hochberg method was applied for false discovery rate (FDR) correction to control the false-positive rate caused by multiple comparisons. In addition, this study further evaluated model stability and sample size adequacy, including the event number, final number of variables included in the model, event per variable (EPV), and concordance index (C-index), in order to comprehensively assess model predictive performance and statistical reliability.

In addition, a supplementary analysis restricted to NEUT-related endpoints was performed to explore the potential influence of granulocyte-colony stimulating factor (G-CSF) exposure. Three endpoint-specific G-CSF exposure labels were generated for grade ≥1 neutropenia, grade ≥3 neutropenia, and severe neutropenia/neutropenia-related complications (NEUT <0.5 × 10^9^/L), respectively. G-CSF exposure was counted only when the first recorded use occurred before the first occurrence of the corresponding endpoint; use on or after endpoint occurrence was not considered pre-endpoint exposure. Time-dependent Cox models including G-CSF as a time-varying covariate were preferentially used, with endpoint-specific static Cox models retained as supplementary sensitivity analyses.

### 2.5 Exploratory dynamic pattern analysis

To further explore whether potential heterogeneity exists in chemotherapy-related myelosuppression and whether different patients exhibit distinct myelosuppression evolution patterns, this study additionally performed exploratory clustering analyses. The primary focus was to determine whether systematic differences existed among patients in terms of myelosuppression severity, fluctuation characteristics, and recovery capacity.

For this purpose, unsupervised clustering analyses were conducted based on the longitudinal features of four key indicators (HGB, NEUT, PLT, and WBC) during chemotherapy. Extracted features mainly included statistical summary features reflecting overall distribution and fluctuation characteristics, including median, mean, variance, standard deviation, minimum value, and maximum value. All features were standardized using the *z*-score method. Subsequently, *K*-means clustering analysis was performed based on the standardized feature matrix, and silhouette scores corresponding to different cluster numbers were compared within the range of *k* = 2 to 10. The optimal number of clusters was determined in combination with clinical interpretability. After clustering, principal component analysis (PCA) was applied for dimensionality reduction and visualization of high-dimensional features to demonstrate the distribution characteristics among different clustering subtypes. Differences among clustering subtypes in terms of myelosuppression occurrence, maximum grade, fluctuation, and remission characteristics were further compared. In addition, differences in baseline clinical characteristics and laboratory parameters among clustering subtypes were analyzed to evaluate their potential predictive value for different myelosuppression patterns. Grade-based HGB and NEUT variables used for post-clustering comparisons were calculated using the WHO thresholds specified in Table 1.

### 2.6 Statistical analysis tools

**Figure 1:**
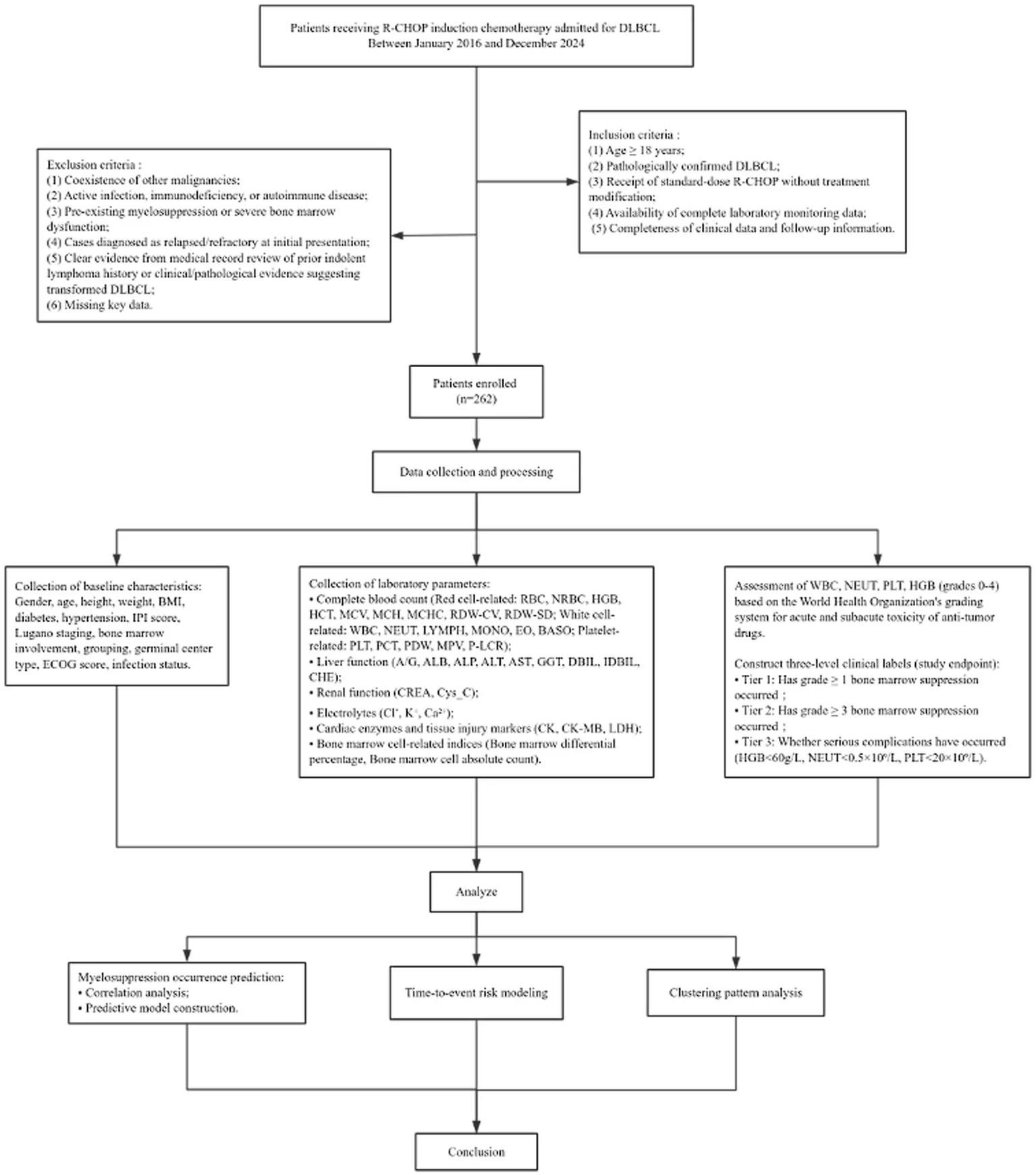
Flowchart of the study design.

All tests were two-sided with significance set at *α* = 0.05. Analyses were conducted using Python (version 3.12.7) on longitudinal chemotherapy data. The study workflow is illustrated in Figure 1.

## 3 Results

### 3.1 Baseline characteristics

A total of 262 patients with DLBCL receiving R-CHOP treatment were included. The median follow-up duration was 270.50 (139.00–771.25) days, with a median of 20.00 (11.25–37.75) follow-up assessments. Among the included patients, 110 were classified as Lugano stage ≤II and 152 as Lugano stage >II. In patients with advanced Lugano stage, age was relatively higher (*p* = 0.011). Significant differences were also observed between the two groups in IPI score distribution, patient subgroup, bone marrow involvement, and germinal center subtype (all *p* < 0.05). No statistically significant differences were observed between the two groups in baseline characteristics including sex, BMI, diabetes mellitus, hypertension, infection status, and ECOG score (all *p* > 0.05). Regarding myelosuppression-related endpoints, patients with advanced Lugano stage were more likely to develop grade ≥3 HGB- and PLT-related myelosuppression events, while the incidences of severe anemia and high infection risk-related neutropenia were also relatively higher (all *p* < 0.05). Grade ≥3 NEUT-related myelosuppression did not differ significantly by Lugano stage (*p* = 0.362). In addition, the incidence of grade 1 PLT-related myelosuppression was significantly increased in the advanced Lugano stage group (*p* = 0.013). Using the single-population proportion sample size estimation method based on event rates, the theoretically required sample sizes for the three endpoint levels ranged from 102 to 196 cases. The actual sample size included in this study was 262 cases for all analyses, which exceeded the corresponding theoretically required sample sizes (see Table 2).

**Table 2:** Characteristics of patients with diffuse large B-cell lymphoma.

Variable	Low-stage group (*n* = 110)	High-stage group (*n* = 152)	*p* value
Age	55.00 (47.25–64.00)	61.00 (52.00–68.00)	0.011
Height	165.00 (160.00–171.00)	165.00 (160.00–170.00)	0.789
Weight	66.50 (56.25–75.00)	65.00 (58.75–72.00)	0.662
BMI	23.98 (21.41–26.57)	23.65 (21.69–26.56)	0.844
Sex			0.243
Male	61 (55.5%)	72 (47.4%)	
Female	49 (44.5%)	80 (52.6%)	
Diabetes mellitus			0.286
No	101 (91.8%)	132 (86.8%)	
Yes	9 (8.2%)	20 (13.2%)	
Hypertension			0.391
No	91 (82.7%)	118 (77.6%)	
Yes	19 (17.3%)	34 (22.4%)	
IPI score			<0.001
0	36 (32.7%)	2 (1.3%)	
1	52 (47.3%)	20 (13.2%)	
2	14 (12.7%)	41 (27.0%)	
3	8 (7.3%)	48 (31.6%)	
4	0 (0.0%)	28 (18.4%)	
5	0 (0.0%)	13 (8.6%)	
Group			<0.001
a	94 (85.5%)	100 (65.8%)	
b	16 (14.5%)	52 (34.2%)	
Germinal center subtype			0.049
No	70 (63.6%)	115 (75.7%)	
Yes	40 (36.4%)	37 (24.3%)	
ECOG score			0.299
0	79 (71.8%)	94 (61.8%)	
1	23 (20.9%)	34 (22.4%)	
2	4 (3.6%)	12 (7.9%)	
3	1 (0.9%)	4 (2.6%)	
4	2 (2.7%)	8 (5.3%)	
5	0 (0.0%)	0 (0.0%)	
Infection			0.485
No	107 (97.3%)	144 (94.7%)	
Yes	3 (2.7%)	8 (5.3%)	
Bone marrow involvement			<0.001
No	110 (100.0%)	116 (76.3%)	
Yes	0 (0.0%)	36 (23.7%)	
Grade 1 HGB myelosuppression			0.604
No	27 (24.5%)	32 (21.1%)	
Yes	83 (75.5%)	120 (78.9%)	
Grade 3 HGB myelosuppression			0.007
No	75 (68.2%)	77 (50.7%)	
Yes	35 (31.8%)	75 (49.3%)	
Severe anemia			0.024
No	91 (82.7%)	106 (69.7%)	
Yes	19 (17.3%)	46 (30.3%)	
Grade 1 WBC myelosuppression			0.806
No	18 (16.4%)	22 (14.5%)	
Yes	92 (83.6%)	130 (85.5%)	
Grade 3 WBC myelosuppression			0.118
No	63 (57.3%)	71 (46.7%)	
Yes	47 (42.7%)	81 (53.3%)	
Grade 1 NEUT myelosuppression			0.328
No	21 (19.1%)	21 (13.8%)	
Yes	89 (80.9%)	131 (86.2%)	
Grade 3 NEUT myelosuppression			0.362
No	49 (44.5%)	58 (38.2%)	
Yes	61 (55.5%)	94 (61.8%)	
Severe neutropenia with high infection risk			0.011
No	78 (70.9%)	83 (54.6%)	
Yes	32 (29.1%)	69 (45.4%)	
Grade 1 PLT myelosuppression			0.013
No	65 (59.1%)	65 (42.8%)	
Yes	45 (40.9%)	87 (57.2%)	
Grade 3 PLT myelosuppression			0.025
No	80 (72.7%)	89 (58.6%)	
Yes	30 (27.3%)	63 (41.4%)	
High bleeding risk			0.086
No	86 (78.2%)	103 (67.8%)	
Yes	24 (21.8%)	49 (32.2%)	

### 3.2 Correlation analysis between baseline clinical characteristics, laboratory parameters, and myelosuppression occurrence

As shown in Supplementary Table 1, in grade 1 PLT-related myelosuppression, Lugano stage and patient subgroup were significantly associated with event occurrence (both *p* < 0.05), with a higher proportion of stage III–IV and high-risk subgroup patients observed in the event group. Grade 3 PLT-related myelosuppression was also associated with patient subgroup (*p* = 0.012). High bleeding risk events (PLT < 20 × 10^9^/L) were associated with patient subgroup and ECOG score (both *p* < 0.05), with severe bleeding events occurring more frequently in patients with high-risk subgroup classification and higher ECOG scores. Grade 1 WBC-related myelosuppression was associated with IPI score, Lugano stage, sex, and body weight (all *p* < 0.05). In the event group, higher proportions of patients with high IPI scores, stage III–IV disease, and female sex were observed, while body weight levels were relatively lower. Grade 3 WBC-related myelosuppression was associated only with patient subgroup (*p* = 0.003). No baseline clinical characteristic was significantly associated with grade ≥1 NEUT-related myelosuppression (all *p* > 0.05). Grade ≥3 NEUT-related myelosuppression was associated with patient subgroup (*p* = 0.015) and infection status (*p* = 0.003), with high-risk subgroup classification and infection occurring more frequently in the event group. Neutropenia-related infection risk (NEUT < 0.5 × 10^9^/L) was also associated with Lugano stage and patient subgroup (both *p* < 0.05). In grade 1 HGB-related myelosuppression, Lugano stage, patient subgroup, sex, height, body weight, and BMI were all significantly associated with event occurrence (all *p* < 0.05). The event group showed higher proportions of stage III–IV disease, high-risk subgroup classification, and female patients, together with lower height, body weight, and BMI levels. In grade 3 myelosuppression, HGB-related events were significantly associated with Lugano stage, patient subgroup, sex, height, and body weight (all *p* ≤ 0.011). The event group demonstrated increased proportions of stage IV disease, high-risk subgroup classification, and female patients, accompanied by lower height and body weight levels. Severe anemia risk (HGB < 60 g/L) was associated with patient subgroup and sex (both *p* < 0.05), with higher proportions of high-risk subgroup patients and female patients observed in the event group.

**Figure 2:**
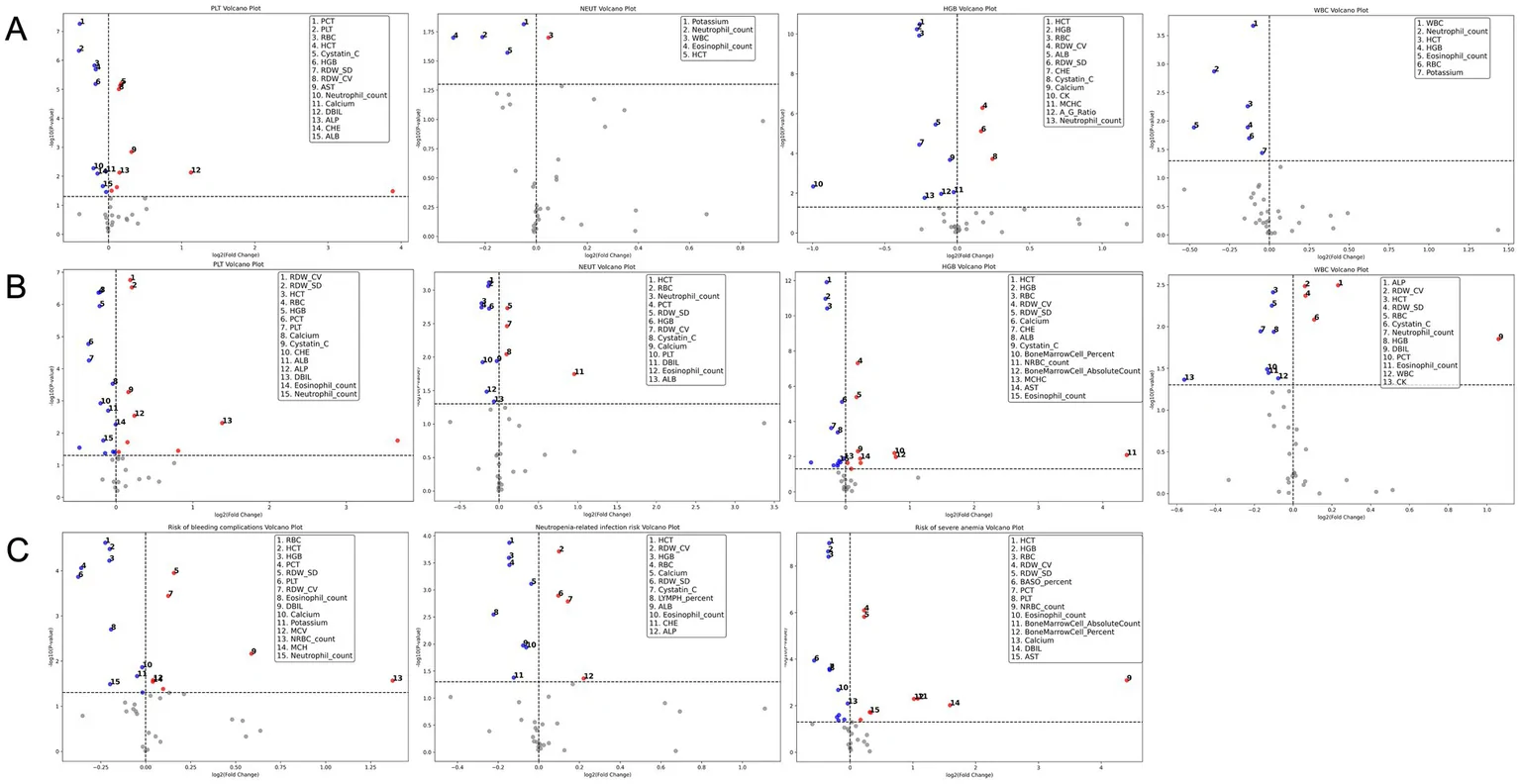
Correlations between baseline characteristics and myelosuppression occurrence. **(A)** Correlations with the occurrence of grade ≥1 myelosuppression; **(B)** Correlations with the occurrence of grade ≥3 myelosuppression; **(C)** Correlations with the occurrence of severe myelosuppression-related complications.

As shown in Figure 2, in grade 1 HGB-related myelosuppression, HCT, HGB, RBC, and ALB were all significantly negatively correlated with myelosuppression occurrence, whereas RDW-CV showed a significant positive correlation. HCT, HGB, RBC, and ALB were also significantly negatively correlated with grade 3 anemia occurrence, whereas RDW-CV, RDW-SD, AST, and ALP showed significant positive correlations. For severe anemia risk (HGB < 60 g/L), HCT, HGB, RBC, PCT, and PLT were all significantly negatively correlated with event occurrence (all *p* < 0.001), whereas RDW-CV, RDW-SD, DBIL, and AST showed significant positive correlations (all *p* < 0.05). Absolute bone marrow cell count and bone marrow cell proportion were also positively correlated with severe anemia (both *p* < 0.01). In grade 1 WBC-related myelosuppression, baseline WBC, NEUT, HCT, HGB, and EO# were all negatively correlated with leukocyte suppression occurrence (all *p* < 0.05). ALP, RDW-CV, and RDW-SD were significantly positively correlated with grade 3 myelosuppression (all *p* < 0.01), whereas HCT and RBC showed significant negative correlations (both *p* < 0.01). In grade ≥1 NEUT-related myelosuppression, K^+^, NEUT#, EO#, and HCT were significantly negatively associated with event occurrence (all *p* < 0.05), whereas WBC showed a positive association (*p* = 0.020). In grade ≥3 NEUT-related myelosuppression, HCT, RBC, NEUT#, PCT, HGB, Ca, PLT, EO#, and ALB were significantly negatively associated with event occurrence, whereas RDW-SD, RDW-CV, Cys-C, and DBIL showed significant positive associations (all *p* < 0.05). For neutropenia-related infection risk (NEUT < 0.5 × 10^9^/L), HCT, HGB, RBC, ALB, Ca, and LYMPH% were all significantly negatively correlated with event occurrence (all *p* < 0.05), whereas RDW-CV, RDW-SD, Cys-C, and ALP showed significant positive correlations (all *p* < 0.05). In the PLT dimension, PCT, PLT, RBC, and HCT were all significantly negatively correlated with platelet suppression occurrence (all *p* < 0.001), whereas Cys-C showed a significant positive correlation (*p* < 0.001). RDW-CV and RDW-SD were significantly positively correlated with grade 3 myelosuppression occurrence (both *p* < 0.001), whereas HCT, RBC, and HGB all showed significant negative correlations (all *p* < 0.001). In addition, ALB was negatively correlated with severe thrombocytopenia (*p* = 0.002), whereas AST and ALP were positively correlated (*p* = 0.019 and *p* = 0.003, respectively). For high bleeding risk (PLT < 20 × 10^9^/L), RBC, HCT, HGB, PCT, and PLT were all significantly negatively correlated with event occurrence (all p < 0.001), whereas RDW-CV, RDW-SD, DBIL, MCV, and MCH showed significant positive correlations (all *p* < 0.05). In addition, NEUT#, EO#, Ca, and K^+^ were negatively correlated with high bleeding risk (all *p* < 0.05).

### 3.3 Machine learning-based prediction of myelosuppression occurrence

**Figure 3:**
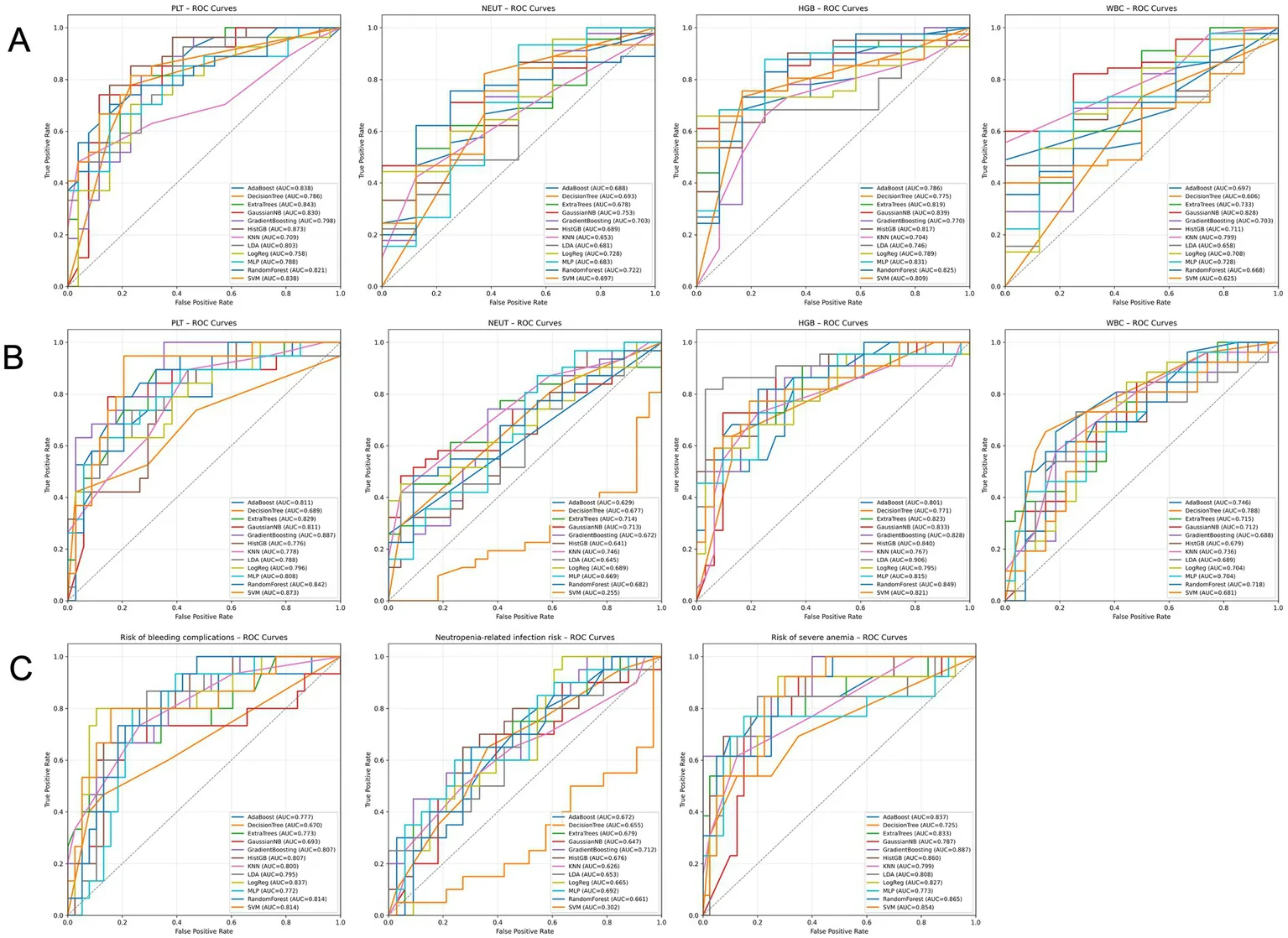
Machine learning results based on baseline characteristics. **(A)** Machine learning models based on grade ≥1 myelosuppression occurrence labels; **(B)** machine learning models based on grade ≥3 myelosuppression occurrence labels; **(C)** machine learning models based on severe myelosuppression-related complication occurrence labels.

As shown in Figure 3, to further transform potential linear correlations into quantitative characterization of complex nonlinear relationships, machine learning models were constructed for multiple dimensions of myelosuppression events. In addition to evaluating the discriminative ability of the models for myelosuppression occurrence, model performance was also used to further assess the predictability of different events. The results showed that the model AUCs for grade 1 myelosuppression, grade 3 myelosuppression, and bleeding risk in the HGB and PLT dimensions were all greater than 0.8, suggesting that these events were less influenced by subsequent treatment processes and were instead strongly affected by baseline bone marrow status. As the severity of myelosuppression increased, the predictive performance of WBC models showed a slight decline. In contrast, predictive performance for the NEUT dimension was relatively lower: the highest mean test-set AUC was 0.606 for grade ≥1 events and 0.614 for grade ≥3 events, indicating that neutropenia was influenced by both treatment-related factors and baseline bone marrow status.

### 3.4 Time-to-event risk analysis of myelosuppression

Based on the previous correlation analyses and machine learning evaluations of myelosuppression predictability, this study further incorporated the time dimension and applied Cox regression models to analyze the occurrence risk of different types and severities of myelosuppression, in order to further quantify the dynamic effects of baseline clinical characteristics and laboratory parameters on event risk. To reduce the false-positive risk caused by multiple comparisons, FDR correction was additionally performed for the above model results. For grade 1 HGB-related myelosuppression (*C*-index: 0.85, EPV: 7.00), baseline HCT was identified as a significant protective factor (HR = 0.918, 95% CI: 0.894–0.943, *p* < 0.001). Similar findings were observed in grade 3 myelosuppression (C-index: 0.83, EPV: 3.93) (HR = 0.910, 95% CI: 0.882–0.940, *p* < 0.001). For severe anemia risk (*C*-index: 0.84, EPV: 2.83), baseline HCT remained a significant protective factor (HR = 0.934, 95% CI: 0.902–0.966, *p* < 0.001). For grade 1 PLT-related myelosuppression (*C*-index: 0.78, EPV: 6.29), baseline RDW-CV acted as a risk factor (HR = 1.113, 95% CI: 1.034–1.198, *p* = 0.005), whereas PCT was identified as a protective factor (HR = 0.005, 95% CI: 0.001–0.043, *p* < 0.001). In grade 3 PLT-related myelosuppression (*C*-index: 0.78, EPV: 3.72), RDW-CV remained significant after FDR correction (HR = 1.161, 95% CI: 1.073–1.255, *p* < 0.001), whereas MCH and PCT showed nominal associations but did not remain significant after FDR correction. For grade ≥1 NEUT-related myelosuppression (220 events; *C*-index: 0.69; EPV: 14.67), baseline NEUT# was a significant protective factor (HR = 0.835, 95% CI: 0.760–0.918; FDR-adjusted *p* = 0.0026). For grade ≥3 NEUT-related myelosuppression (155 events; *C*-index: 0.67; EPV: 8.61), no covariate remained significant after FDR correction. For neutropenia-related infection risk (101 events; *C*-index: 0.68; EPV: 5.32), baseline DBIL remained a significant risk factor (HR = 1.043, 95% CI: 1.029–1.057; FDR-adjusted *p* < 0.001). In grade 1 WBC-related myelosuppression (*C*-index: 0.72, EPV: 15.86), baseline NEUT was identified as a significant protective factor (HR = 0.837, 95% CI: 0.769–0.911, *p* < 0.001). No significant factors were identified for the remaining endpoints.

In the supplementary NEUT-specific G-CSF analysis, 149 of 262 patients had recorded G-CSF exposure. The numbers of patients exposed before the corresponding endpoint or censoring time were 61 for grade ≥1 neutropenia, 92 for grade ≥3 neutropenia, and 116 for severe neutropenia. In time-dependent Cox models adjusted for the corresponding baseline predictors, G-CSF(t) was not significantly associated with grade ≥1 neutropenia (HR = 0.94, 95% CI: 0.67–1.31, *p* = 0.700). Associations with grade ≥3 neutropenia (HR = 1.45, 95% CI: 1.03–2.03, *p* = 0.031) and severe neutropenia (HR = 1.67, 95% CI: 1.14–2.44, *p* = 0.008) did not remain statistically significant after endpoint-level false discovery rate correction.

### 3.5 Exploratory pattern analysis of myelosuppression

**Figure 4:**
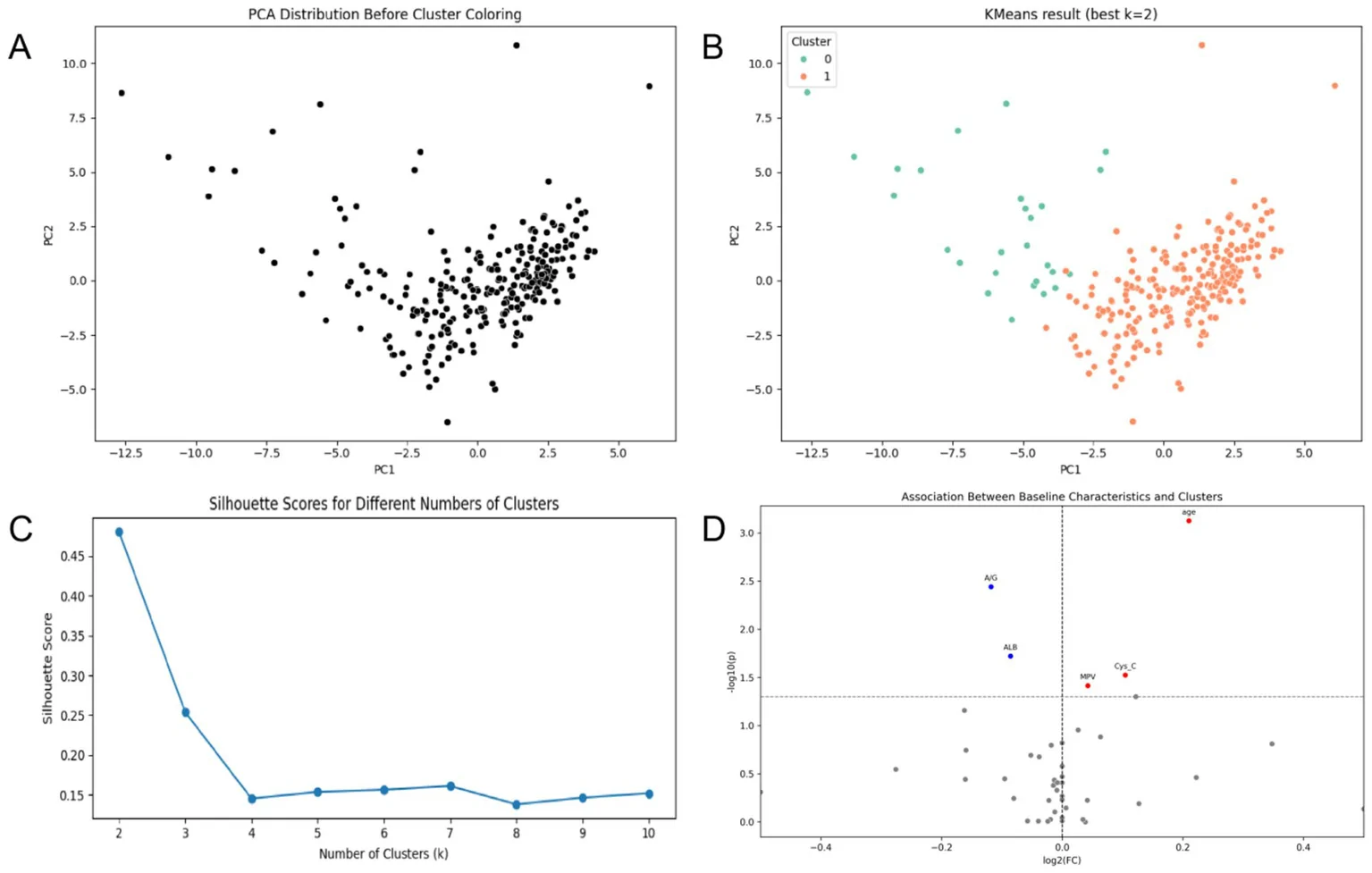
Clustering analysis results. **(A)** Distribution of the original data after dimensionality reduction; **(B)** distribution of the data after clustering; **(C)** line plot of silhouette scores across different numbers of clusters; **(D)** volcano plot showing correlations between subtypes and baseline characteristics.

After evaluating the risk of myelosuppression occurrence, this study further explored potential heterogeneity during the evolution of myelosuppression. As shown in Figure 4, clustering analysis classified 27 patients into subtype 1 and 235 patients into subtype 2. Subtype 1 was characterized by greater severity and longitudinal variability across the HGB, WBC, NEUT, and PLT dimensions. In the HGB dimension, subtype 1 showed higher event occurrence (96.3% vs. 75.3%, *p* = 0.026), more frequent fluctuation events (96.3% vs. 73.2%, *p* = 0.016), more frequent remission events (77.8% vs. 44.3%, *p* = 0.002), and a higher mean maximum grade (3.52 vs. 1.87, *p* < 0.001). It also had a lower longitudinal mean and minimum HGB value and substantially greater variance and standard deviation. In the NEUT dimension, subtype 1 showed higher event occurrence (100.0% vs. 82.1%, *p* = 0.034) and a higher mean maximum grade (3.81 vs. 2.40, *p* < 0.001), together with markedly greater variance and standard deviation; differences in NEUT fluctuation and remission events were not statistically significant. In the PLT dimension, subtype 1 remained positively associated with PLT-related events and remission events (both *p* < 0.001). Subtype 2 therefore represented a comparatively milder and more stable pattern rather than a uniformly more recoverable pattern. Compared with subtype 1, patients in subtype 2 were older (*p* = 0.003) and had higher baseline Cys-C (*p* = 0.010) and MPV levels (*p* = 0.027), whereas subtype 1 exhibited higher baseline A/G (*p* = 0.001) and ALB levels (*p* = 0.002). No statistically significant differences were observed in the remaining baseline indicators between the two groups (all *p* > 0.05). G-CSF use was numerically more frequent in subtype 1 than in subtype 2 (70.4% vs. 55.3%). However, this difference did not reach statistical significance (Pearson *χ*^2^ = 2.24, *p* = 0.135), suggesting that G-CSF exposure was not significantly imbalanced between the two dynamic myelosuppression subtypes.

## 4 Discussion

This study systematically analyzed R-CHOP–related myelosuppression in DLBCL patients from four perspectives: baseline correlations, predictive performance, temporal risk, and dynamic heterogeneity. Analyses of baseline clinical characteristics and laboratory parameters demonstrated that different types of myelosuppression did not share a unified pathological basis. HGB- and PLT-related myelosuppression appeared to depend more strongly on pre-existing bone marrow status, whereas NEUT- and WBC-related abnormalities were more susceptible to chemotherapy toxicity, inflammatory responses, and dynamic alterations in the bone marrow microenvironment. Cox regression analysis further revealed lineage-specific differences in the composition of risk factors across different myelosuppression events. Unsupervised *K*-means clustering based on longitudinal hematologic features additionally identified a high-severity, high-variability pattern and a comparatively mild, stable pattern; recovery-related indicators were dimension-specific and did not support a uniform poor-recovery versus recoverable dichotomy.

This study first systematically analyzed the occurrence patterns of myelosuppression in DLBCL patients following R-CHOP treatment from the perspective of baseline clinical characteristics. Different types of myelosuppression exhibited distinct association patterns at the baseline clinical level. Due to the relatively slow erythropoietic process and the long lifespan of erythrocytes, alterations in the erythroid lineage are more likely to reflect the long-term cumulative hematopoietic status of patients. Therefore, in patients with higher tumor burden, more pronounced chronic consumption, or poorer baseline nutritional status, the erythroid system may enter a low-reserve state earlier, thereby becoming more susceptible to persistent anemia and severe anemia during chemotherapy (27)^.^ In addition, the association between germinal center subtype and HGB-related myelosuppression suggests that tumor behaviors under different molecular biological backgrounds may indirectly affect erythroid stability through alterations in inflammatory status, bone marrow infiltration tendency, or metabolic consumption (28). The leukocyte system itself undergoes relatively rapid turnover and is more sensitive to chemotherapeutic agents, infection, and inflammatory stimulation; therefore, severe leukocyte suppression may more strongly reflect acute stress effects during treatment. Accordingly, in the present study, the leukocyte system was influenced by baseline disease status mainly during the early stage of disease progression. No baseline clinical characteristic was associated with grade ≥1 NEUT-related myelosuppression, whereas grade ≥3 events were associated with patient subgroup and infection status. Neutropenia-related infection risk remained associated with Lugano stage and patient subgroup. These findings suggest that clinical determinants differ according to the severity and clinical consequence of neutrophil suppression (29, 30). The PLT dimension was more closely associated with overall functional status and disease progression. This may be because the platelet system is influenced not only by bone marrow megakaryopoietic capacity, but also by tumor-related inflammatory status, systemic consumption, and overall functional condition. Patients with poorer functional status are often accompanied by more pronounced systemic frailty, inflammatory activation, or coagulation homeostasis abnormalities, making them more susceptible to severe thrombocytopenia and bleeding events following chemotherapy (31)^.^

When temporal factors were taken into consideration, insufficient erythroid hematopoietic reserve and increased erythrocyte heterogeneity were identified as important foundations for the development of anemia in HGB-related myelosuppression. Meanwhile, stress erythropoiesis, ineffective hematopoiesis, and chronic inflammatory states were associated with the persistence of severe anemia (32). In addition, nutritional status, hepatic metabolic function, and bilirubin metabolism abnormalities may contribute to anemia development through effects on iron metabolism, EPO regulation, and chronic inflammatory responses. In PLT-related myelosuppression, megakaryocytic production capacity and overall bone marrow reserve played central roles in maintaining platelet homeostasis. At the same time, the findings suggested that abnormal erythropoietic states may coexist with impaired megakaryopoiesis. Furthermore, liver dysfunction may contribute to bleeding risk through alterations in coagulation factor synthesis, splenic compensation, and platelet clearance. Thrombocytopenia may therefore reflect not only direct megakaryocyte injury, but also systemic inflammatory status, electrolyte imbalance, and impaired vascular endothelial stability (33). The WBC and NEUT dimensions more strongly reflected the sensitivity of the granulocytic system to inflammation and dynamic stress responses. In WBC-related myelosuppression, relatively preserved baseline hematopoietic reserve appeared beneficial for maintaining leukocyte homeostasis, whereas systemic inflammation, metabolic abnormalities, and tissue injury may contribute to severe leukocyte suppression. In NEUT-related myelosuppression, a higher baseline NEUT# was independently associated with a lower hazard of grade ≥1 events, whereas no baseline covariate remained significant for grade ≥3 events after FDR correction. By contrast, DBIL remained independently associated with neutropenia-related infection risk. These findings support a distinction between susceptibility to early neutrophil suppression and the risk of clinically severe neutropenia-related complications. Machine learning models further supported these findings, suggesting that HGB- and PLT-related events were more strongly determined by pre-existing hematopoietic reserve and baseline bone marrow status. In contrast, granulocyte-related injury appeared to be more susceptible to treatment-related factors, including chemotherapy exposure, infection, and dynamic inflammatory responses. The supplementary G-CSF analysis further supports the interpretation that the primary models should be viewed as baseline prognostic models rather than treatment-response models.

Because myelosuppression often imposes a direct psychological burden on patients, we conducted an exploratory analysis to determine whether distinct dynamic patterns could be identified. The results demonstrated that myelosuppression should not be regarded merely as a binary event defined by occurrence or non-occurrence, but rather as a process characterized by substantial heterogeneity (27)^.^ Subtype 1 exhibited higher maximum grades and markedly greater variability across hematopoietic dimensions. It also showed more frequent HGB and NEUT events and more frequent HGB fluctuations. However, HGB remission events were also more frequent in subtype 1, while NEUT remission did not differ significantly between subtypes. Thus, subtype 1 is more appropriately interpreted as a high-severity, high-instability pattern rather than a uniformly poor-recovery pattern. Subtype 2 demonstrated milder suppression, smaller fluctuation ranges, and higher longitudinal HGB levels, supporting its interpretation as a comparatively stable pattern without assuming superior recovery across every hematologic lineage. The older age and higher MPV and Cys-C levels in subtype 2, together with higher ALB and A/G levels in subtype 1, further suggest that these patterns reflect integrated differences in physiological reserve, organ compensation, and treatment-related dynamics rather than nutritional status alone (34–38). This finding suggests that the more severe and variable myelosuppression pattern observed in subtype 1 is unlikely to be explained solely by an imbalance in G-CSF exposure between subtypes. Therefore, the subtype differences may more plausibly reflect underlying heterogeneity in hematopoietic reserve, treatment sensitivity, and systemic physiological status rather than differential use of granulocyte-supportive therapy alone.

Nevertheless, this study has several limitations. First, the study design may introduce selection bias (e.g., differences in patient baseline characteristics) and provides limited control over confounding factors. Second, the single-center nature of the data limits generalizability; regional population differences may affect external validity of the conclusions. Furthermore, causal relationships between laboratory indices and myelosuppression remain to be established. Because follow-up data between two chemotherapy cycles could not be obtained at completely regular intervals, potential sampling bias may exist. Additionally, the sample size for some Cox analyses was insufficient; therefore, these analyses could only be considered exploratory in this study, and further validation with expanded datasets is needed. Future work should undertake multicenter prospective cohort studies to validate the predictive performance of identified biomarkers and the stability of dynamic patterns, thereby facilitating clinical translation and supporting precision medical decision-making.

## 5 Conclusion

This study systematically evaluated the occurrence patterns, risk characteristics, and dynamic heterogeneity of different types of myelosuppression in DLBCL patients during R-CHOP treatment. Myelosuppression involving different hematopoietic lineages exhibited substantial differences in clinical characteristic associations, laboratory parameter composition, and predictive performance. Furthermore, myelosuppression should not be considered a single homogeneous process, but rather comprises a high-severity, high-variability subtype and a comparatively mild and stable subtype, with recovery-related characteristics varying across hematologic lineages. These findings provide new evidence for precise risk stratification, dynamic monitoring, and individualized intervention for myelosuppression in DLBCL patients.

## Data Availability

The original contributions presented in the study are included in the article/Supplementary material, further inquiries can be directed to the corresponding authors.

## Glossary

R-CHOP: rituximab + cyclophosphamide, doxorubicin, vincristine, prednisone
DLBCL: diffuse large B-cell lymphoma
HGB: hemoglobin
NEUT: neutrophil count
PLT: platelet count
WBC: white blood cell count
PCA: principal component analysis
NHL: non-Hodgkin lymphoma
DNA: deoxyribonucleic acid
G-CSF: granulocyte-colony stimulating factor
HR: hazard ratio
95% CI: 95% confidence interval
RDI: relative dose intensity
CBC: complete blood count
RBC: red blood cell count
NRBC: nucleated red blood cell count
HCT: hematocrit
MCV: mean corpuscular volume
MCH: mean corpuscular hemoglobin
MCHC: mean corpuscular hemoglobin concentration
RDW-CV: red cell distribution width-coefficient of variation
RDW-SD: red cell distribution width-standard deviation
LYMPH: lymphocyte count
MONO: monocyte count
EO: eosinophil count
BASO: basophil count
PCT: plateletcrit
PDW: platelet distribution width
MPV: mean platelet volume
P-LCR: platelet-large cell ratio
RET: reticulocyte count
RET-He: reticulocyte hemoglobin equivalent
IRF: immature reticulocyte fraction
LFR: low-fluorescence ratio
MFR: medium-fluorescence ratio
HFR: high-fluorescence ratio
A/G: albumin/globulin ratio
ALB: albumin
ALP: alkaline phosphatase
ALT: alanine aminotransferase
AST: aspartate aminotransferase
GGT: gamma-glutamyl transferase
DBIL: direct bilirubin
IDBIL: indirect bilirubin
GLB: globulin
CHE: cholinesterase
CREA: creatinine
CREA-eGFR: estimated glomerular filtration rate by creatinine
Cys_C: cystatin C
eGFR1: first method of estimated glomerular filtration rate
eGFR2: second method of estimated glomerular filtration rate
Cl^−^: chloride
K^+^: potassium
Ca: calcium
CHOL: total cholesterol
HDL-C: high-density lipoprotein cholesterol
LDL-C: low-density lipoprotein cholesterol
GLU0: fasting glucose
CK: creatine kinase
CK-MB: creatine kinase-MB isoenzyme
LDH: lactate dehydrogenase
C1q: complement component 1q
WHO: The World Health Organization
RCS: restricted cubic spline
SE: standard error
DBI: Davies–Bouldin index
CNS: central nervous system
EPO: erythropoietin
