# Supplementary material for: Early identification of high-risk myelosuppression subtypes in DLBCL patients undergoing R-CHOP chemotherapy
Source: Front Med (Lausanne). 2026 Aug 18;13:1839446. doi: 10.3389/fmed.2026.1839446 (PMC13529567; doi:10.3389/fmed.2026.1839446)
Supplement: Supplementary file 1 [file Table_1.DOCX]

**SM Table 1 Correlations Between Clinical Characteristics and Myelosuppression**

| **LabelGroup** | **Label** | **Feature** | **Category** | **Group0** | **Group1** | **P** | **Method** |
| --- | --- | --- | --- | --- | --- | --- | --- |
| Grade1 | PLT | Lugano stage | 1 | 24 (18.5%) | 14 (10.6%) | 0.038 | Chi-square |
| Grade1 | PLT | Lugano stage | 2 | 41 (31.5%) | 31 (23.5%) | 0.038 | Chi-square |
| Grade1 | PLT | Lugano stage | 3 | 19 (14.6%) | 33 (25.0%) | 0.038 | Chi-square |
| Grade1 | PLT | Lugano stage | 4 | 46 (35.4%) | 54 (40.9%) | 0.038 | Chi-square |
| Grade1 | PLT | Subgroup | 0 | 105 (80.8%) | 89 (67.4%) | 0.017 | Fisher |
| Grade1 | PLT | Subgroup | 1 | 25 (19.2%) | 43 (32.6%) | 0.017 | Fisher |
| Grade1 | PLT | ECOG score | 0 | 87 (66.9%) | 86 (65.2%) | 0.034 | Chi-square |
| Grade1 | PLT | ECOG score | 1 | 31 (23.8%) | 26 (19.7%) | 0.034 | Chi-square |
| Grade1 | PLT | ECOG score | 2 | 2 (1.5%) | 14 (10.6%) | 0.034 | Chi-square |
| Grade1 | PLT | ECOG score | 3 | 3 (2.3%) | 2 (1.5%) | 0.034 | Chi-square |
| Grade1 | PLT | ECOG score | 4 | 7 (5.4%) | 4 (3.0%) | 0.034 | Chi-square |
| Grade1 | WBC | IPI score | 0 | 5 (12.5%) | 33 (14.9%) | 0.026 | Chi-square |
| Grade1 | WBC | IPI score | 1 | 12 (30.0%) | 60 (27.0%) | 0.026 | Chi-square |
| Grade1 | WBC | IPI score | 2 | 6 (15.0%) | 49 (22.1%) | 0.026 | Chi-square |
| Grade1 | WBC | IPI score | 3 | 7 (17.5%) | 49 (22.1%) | 0.026 | Chi-square |
| Grade1 | WBC | IPI score | 4 | 10 (25.0%) | 18 (8.1%) | 0.026 | Chi-square |
| Grade1 | WBC | IPI score | 5 | 0 (0.0%) | 13 (5.9%) | 0.026 | Chi-square |
| Grade1 | WBC | Lugano stage | 1 | 11 (27.5%) | 27 (12.2%) | 0.033 | Chi-square |
| Grade1 | WBC | Lugano stage | 2 | 7 (17.5%) | 65 (29.3%) | 0.033 | Chi-square |
| Grade1 | WBC | Lugano stage | 3 | 5 (12.5%) | 47 (21.2%) | 0.033 | Chi-square |
| Grade1 | WBC | Lugano stage | 4 | 17 (42.5%) | 83 (37.4%) | 0.033 | Chi-square |
| Grade1 | WBC | Sex | 0 | 13 (32.5%) | 120 (54.1%) | 0.016 | Fisher |
| Grade1 | WBC | Sex | 1 | 27 (67.5%) | 102 (45.9%) | 0.016 | Fisher |
| Grade1 | WBC | Weight |  | 71.05 ± 13.31 | 65.00 (58.00–72.00) | 0.022 | Kruskal-Wallis |
| Grade1 | HGB | Lugano stage | 1 | 16 (27.1%) | 22 (10.8%) | 0.012 | Chi-square |
| Grade1 | HGB | Lugano stage | 2 | 11 (18.6%) | 61 (30.0%) | 0.012 | Chi-square |
| Grade1 | HGB | Lugano stage | 3 | 12 (20.3%) | 40 (19.7%) | 0.012 | Chi-square |
| Grade1 | HGB | Lugano stage | 4 | 20 (33.9%) | 80 (39.4%) | 0.012 | Chi-square |
| Grade1 | HGB | Subgroup | 0 | 51 (86.4%) | 143 (70.4%) | 0.017 | Fisher |
| Grade1 | HGB | Subgroup | 1 | 8 (13.6%) | 60 (29.6%) | 0.017 | Fisher |
| Grade1 | HGB | Sex | 0 | 20 (33.9%) | 113 (55.7%) | 0.005 | Fisher |
| Grade1 | HGB | Sex | 1 | 39 (66.1%) | 90 (44.3%) | 0.005 | Fisher |
| Grade1 | HGB | Height |  | 170.00 (162.00–173.50) | 164.00 (160.00–170.00) | 0.003 | Kruskal-Wallis |
| Grade1 | HGB | Weight |  | 72.37 ± 12.57 | 65.00 (56.50–72.00) | <0.001 | Kruskal-Wallis |
| Grade1 | HGB | BMI |  | 24.80 (22.30–26.66) | 23.51 (21.26–26.50) | 0.021 | Kruskal-Wallis |
| Grade3 | HGB | Lugano stage | 1 | 29 (19.1%) | 9 (8.2%) | 0.011 | Chi-square |
| Grade3 | HGB | Lugano stage | 2 | 46 (30.3%) | 26 (23.6%) | 0.011 | Chi-square |
| Grade3 | HGB | Lugano stage | 3 | 30 (19.7%) | 22 (20.0%) | 0.011 | Chi-square |
| Grade3 | HGB | Lugano stage | 4 | 47 (30.9%) | 53 (48.2%) | 0.011 | Chi-square |
| Grade3 | HGB | Subgroup | 0 | 127 (83.6%) | 67 (60.9%) | <0.001 | Fisher |
| Grade3 | HGB | Subgroup | 1 | 25 (16.4%) | 43 (39.1%) | <0.001 | Fisher |
| Grade3 | HGB | Sex | 0 | 62 (40.8%) | 71 (64.5%) | <0.001 | Fisher |
| Grade3 | HGB | Sex | 1 | 90 (59.2%) | 39 (35.5%) | <0.001 | Fisher |
| Grade3 | HGB | Height |  | 168.00 (160.00–173.00) | 163.00 (160.00–168.75) | 0.003 | Kruskal-Wallis |
| Grade3 | HGB | Weight |  | 69.50 (60.00–75.00) | 64.00 (56.25–70.75) | 0.004 | Kruskal-Wallis |
| Grade3 | WBC | Subgroup | 0 | 110 (82.1%) | 84 (65.6%) | 0.003 | Fisher |
| Grade3 | WBC | Subgroup | 1 | 24 (17.9%) | 44 (34.4%) | 0.003 | Fisher |
| Grade3 | NEUT | Subgroup | 0 | 88 (82.2%) | 106 (68.4%) | 0.015 | Fisher |
| Grade3 | NEUT | Subgroup | 1 | 19 (17.8%) | 49 (31.6%) | 0.015 | Fisher |
| Grade3 | NEUT | Infection | 0 | 107 (100.0%) | 144 (92.9%) | 0.003 | Fisher |
| Grade3 | NEUT | Infection | 1 | 0 (0.0%) | 11 (7.1%) | 0.003 | Fisher |
| Grade3 | PLT | Subgroup | 0 | 134 (79.3%) | 60 (64.5%) | 0.012 | Fisher |
| Grade3 | PLT | Subgroup | 1 | 35 (20.7%) | 33 (35.5%) | 0.012 | Fisher |
| SevereEvents | Risk of severe anemia | Subgroup | 0 | 154 (78.2%) | 40 (61.5%) | 0.014 | Fisher |
| SevereEvents | Risk of severe anemia | Subgroup | 1 | 43 (21.8%) | 25 (38.5%) | 0.014 | Fisher |
| SevereEvents | Risk of severe anemia | Sex | 0 | 91 (46.2%) | 42 (64.6%) | 0.010 | Fisher |
| SevereEvents | Risk of severe anemia | Sex | 1 | 106 (53.8%) | 23 (35.4%) | 0.010 | Fisher |
| SevereEvents | Neutropenia-related infection risk | Lugano stage | 1 | 29 (18.0%) | 9 (8.9%) | 0.026 | Chi-square |
| SevereEvents | Neutropenia-related infection risk | Lugano stage | 2 | 49 (30.4%) | 23 (22.8%) | 0.026 | Chi-square |
| SevereEvents | Neutropenia-related infection risk | Lugano stage | 3 | 25 (15.5%) | 27 (26.7%) | 0.026 | Chi-square |
| SevereEvents | Neutropenia-related infection risk | Lugano stage | 4 | 58 (36.0%) | 42 (41.6%) | 0.026 | Chi-square |
| SevereEvents | Neutropenia-related infection risk | Subgroup | 0 | 130 (80.7%) | 64 (63.4%) | 0.002 | Fisher |
| SevereEvents | Neutropenia-related infection risk | Subgroup | 1 | 31 (19.3%) | 37 (36.6%) | 0.002 | Fisher |
| SevereEvents | Risk of bleeding complications | Subgroup | 0 | 147 (77.8%) | 47 (64.4%) | 0.040 | Fisher |
| SevereEvents | Risk of bleeding complications | Subgroup | 1 | 42 (22.2%) | 26 (35.6%) | 0.040 | Fisher |
| SevereEvents | Risk of bleeding complications | ECOG score | 0 | 126 (66.7%) | 47 (64.4%) | 0.019 | Chi-square |
| SevereEvents | Risk of bleeding complications | ECOG score | 1 | 45 (23.8%) | 12 (16.4%) | 0.019 | Chi-square |
| SevereEvents | Risk of bleeding complications | ECOG score | 2 | 6 (3.2%) | 10 (13.7%) | 0.019 | Chi-square |
| SevereEvents | Risk of bleeding complications | ECOG score | 3 | 3 (1.6%) | 2 (2.7%) | 0.019 | Chi-square |
| SevereEvents | Risk of bleeding complications | ECOG score | 4 | 9 (4.8%) | 2 (2.7%) | 0.019 | Chi-square |
